# MFGAN: Multimodal Fusion for Industrial Anomaly Detection Using Attention-Based Autoencoder and Generative Adversarial Network

**DOI:** 10.3390/s24020637

**Published:** 2024-01-19

**Authors:** Xinji Qu, Zhuo Liu, Chase Q. Wu, Aiqin Hou, Xiaoyan Yin, Zhulian Chen

**Affiliations:** 1 School of Information Science and Technology, Northwest University, Xi’an 710127, China; 202233411@stumail.nwu.edu.cn (X.Q.); 2016118100@stumail.nwu.edu.cn (Z.L.); houaiqin@nwu.edu.cn (A.H.); yinxy@nwu.edu.cn (X.Y.); chenzhulian@stumail.nwu.edu.cn (Z.C.); 2 Department of Data Science, New Jersey Institute of Technology, Newark, NJ 07102, USA

**Keywords:** industrial anomaly detection, multimodal fusion, attention-based autoencoder, generative adversarial network

## Abstract

Anomaly detection plays a critical role in ensuring safe, smooth, and efficient operation of machinery and equipment in industrial environments. With the wide deployment of multimodal sensors and the rapid development of Internet of Things (IoT), the data generated in modern industrial production has become increasingly diverse and complex. However, traditional methods for anomaly detection based on a single data source cannot fully utilize multimodal data to capture anomalies in industrial systems. To address this challenge, we propose a new model for anomaly detection in industrial environments using multimodal temporal data. This model integrates an attention-based autoencoder (AAE) and a generative adversarial network (GAN) to capture and fuse rich information from different data sources. Specifically, the AAE captures time-series dependencies and relevant features in each modality, and the GAN introduces adversarial regularization to enhance the model’s ability to reconstruct normal time-series data. We conduct extensive experiments on real industrial data containing both measurements from a distributed control system (DCS) and acoustic signals, and the results demonstrate the performance superiority of the proposed model over the state-of-the-art TimesNet for anomaly detection, with an improvement of 5.6% in F1 score.

## 1. Introduction

In today’s industrial environments, large-scale temporal data play an increasingly important role in the industrial production process. How to use such data to ensure the safe and efficient operation of industrial equipment determines the future core competitiveness and sustainability of enterprises.

### 1.1. Research Background

An anomaly in industry usually refers to an event where the data generated in the industrial production process is inconsistent with normal operation. Such an anomaly is often caused by equipment failure, improper operation, or other external disturbances, and the presence of anomalous data often indicates the existence of safety hazards. Anomaly detection helps factories detect abnormalities in the production process in a timely manner, thus avoiding production accidents and improving production efficiency and product quality.

In the industrial field, anomaly detection has been widely practiced in various production processes. For example, in a manufacturing unit, we need to quickly detect abnormalities in the production line, such as machine failure, negligent operation, etc., and take timely measures to repair and avoid production line downtime or product quality decline. Anomaly detection has also been applied to product quality monitoring and troubleshooting. Quality monitoring is focused on monitoring the stability and consistency of product quality while quality troubleshooting attempts to quickly locate the cause of the problem, reduce troubleshooting time and cost, and improve equipment reliability and productivity.

Particularly, with the rapid development of artificial intelligence and big data technologies, anomaly detection has evolved at an unprecedented pace, proliferating into numerous applications in industrial environments.

### 1.2. Motivations

The research in this paper is motivated in several aspects as follows:

(1) Recently, with the emergence of transformers [1] and attention mechanisms, such techniques have also been explored for anomaly detection. However, the work on integrating attention mechanisms with an autoencoder (AE) is still limited. Current studies predominantly apply a transformer or its variants directly [2,3,4], overlooking the untapped potential of attention mechanisms within AE. Particularly, when handling temporal data, conventional AEs exhibit limitations in capturing crucial contextual information. Introducing attention mechanisms in such scenarios is considered a promising approach, aiming to address the shortcomings of AEs in feature extraction by enabling the model to focus more on pivotal segments within sequences, thereby enhancing the model’s comprehension and modeling capabilities across diverse data modalities. Such integration of attention mechanisms and AE holds promise for groundbreaking advancements in the realm of anomaly detection. Hence, one of the motivations behind this study is to explore how attention mechanisms can enhance the performance of AE in anomaly detection, addressing their limitations in extracting essential sequence information.
(2) Traditional methods for anomaly detection mainly use unimodal data from a single type of sensor or device. However, with the advent of Industry 4.0 and the rapid development of Internet of Things (IoT), the data generated in modern industrial production has become increasingly diverse and complex. Particularly, in the framework of Industry 4.0, the Industrial Internet provides us with real-time, high-throughput, multi-source time-series data. Such data come from a variety of sensors, devices, and production systems, and include multiple modalities such as temperature, pressure, vibration, current, acoustic signal, and images. However, due to the sheer volume and complexity of multimodal data, traditional methods for anomaly detection using a single data source cannot fully utilize such data and are often unable to respond promptly. In recent years, with the rise of cross-domain data fusion, multimodal learning has been widely used to integrate information in multiple modalities from multiple sources to improve the performance and effectiveness of learning models for various tasks. However, for anomaly detection, multimodal learning is considered mainly in the image domain, such as multimodal anomaly detection for 3D point clouds and RGB images [5]. Therefore, another motivation behind this study is to address the research gap in utilizing multimodal data for time-series anomaly detection in real industrial settings. Towards this goal, we propose a novel multimodal learning approach to anomaly detection for the core equipment of cement production, namely, roller press.

To evaluate the performance of the proposed method in real-life industrial settings, we collect data and run experiments in a production environment. Presently, several challenges hinder the acquisition of multimodal data for anomaly detection in real industrial environments. Primarily, there is a lack of anomaly detection datasets that encompass multimodal data, impeding experimental validation. Furthermore, existing time-series anomaly detection datasets are predominantly artificially generated fault data, lacking genuine industrial environment characteristics. Consequently, conducting experiments on such datasets may not adequately reflect the effectiveness of the model in practice. Even if our model performs well on artificially generated datasets, it cannot guarantee a similar performance in real environments. Additionally, the privacy concerns surrounding large-scale specialized equipment in the industrial IoT make it challenging for researchers to access their monitoring time-series data, let alone their anomalous data. This difficulty significantly exacerbates the acquisition of authentic anomaly data.

In this work, we collect and organize multimodal anomaly detection data in real industrial environments to assess the reliability and practicality of the proposed model for anomaly detection in collaboration with a third-party cement factory.

### 1.3. Contributions

In the modern industrial landscape, anomaly detection remains a critical task and also a significant challenge. We propose a method that combines an attention mechanism with an AE and a multimodal feature fusion module, and validate the effectiveness of this solution in a real industrial environment. The contributions of our work in this paper are summarized as follows:

(1) We propose a method that utilizes attention mechanisms in an AE, which extracts the sequence context information from the original temporal data and improves the model’s feature extraction for each modal data point.
(2) We design a multimodal feature fusion module, which fuses multiple features such as temperature, vibration, and acoustic signal to improve the accuracy of anomaly detection. This module improves the model’s generalization ability, robustness, and applicability to a wide spectrum of practical scenarios.
(3) We collect multimodal data in a real-life industrial environment and experimentally demonstrate the performance superiority of the proposed model over existing methods for anomaly detection.

The rest of this paper is organized as follows. In Section 2, we conduct a survey of related work. In Section 3, we propose MFGAN for anomaly detection and elaborate the attention-based autoencoder (AAE) and feature fusion module. In Section 4, we present the dataset used in the experiments, model parameters, and experimental results. In Section 5, we summarize our work and sketch a plan of future research.

## 2. Related Work

Methods for anomaly detection can be divided into two categories: unimodal and multimodal methods. Most traditional methods for anomaly detection are based on unimodal data, as evidenced in many domains including cybersecurity and biomedicine. Unimodal methods mainly rely on a single data source or feature for anomaly detection, which means that they only use information from a single data distribution or a specific set of features. In contrast, multimodal methods utilize information from multiple data sources or multiple feature sets for anomaly detection. Unimodal approaches may work well for specific datasets or specific anomaly types, but may suffer from low accuracy when dealing with complex real-world data. Multimodal approaches, on the other hand, are able to capture the correlation and complexity between data more comprehensively, leading to better identification of anomalous behaviors or data points. By combining information from different data sources or feature sets, multimodal approaches can provide more comprehensive and robust detection capabilities to cope with diverse and dynamic anomalies.

### 2.1. Traditional Methods for Anomaly Detection

Traditional methods for anomaly detection can be categorized into the following four types.

(1) **Density estimation-based methods.** Local outlier factor (LOF) [6] is a widely used outlier detection algorithm, while clustering with outlier factor (COF) [7] introduces the concept of connectivity based on LOF, which allows the algorithm to handle high-dimensional data and different types of data. DAGMM [8] combines a Gaussian mixture model (GMM) [9] with neural networks for anomaly detection.
(2) **Reconstruction-based methods.** These methods use normal data to train a model and detect anomalies by comparing the original data with the reconstructed data. Park et al. [10] proposed an LSTM-VAE model, which uses long short-term memory (LSTM) [11] to extract features from the original temporal data and uses a variational autoencoder (VAE) [12] to reconstruct the hidden features. Su et al. [13] also proposed a VAE model, but they utilized a gated recurrent unit (GRU) [14] to extract latent features. TimesNet [15] employs a fast Fourier transformation to convert time-series data from one-dimensional to two-dimensional, and uses classical algorithms in computer vision for anomaly detection. In addition, generative adversarial networks (GANs) [16] have also been applied to anomaly detection. AnoGAN [17] uses a GAN for anomaly detection for the first time and it uses a simple DCGAN [18] structure. It learns to generate normal images by feeding noise vectors to a generator built by an inverse convolutional layer. However, AnoGAN still needs to update its parameters during the testing phase. To address this shortcoming, Zenati et al. [19] proposed a BiGAN-based [20] approach, which greatly accelerates the processing speed of the model by adding an encoder that maps the input sample to the potential representation during training. f-AnoGAN [21], on the other hand, trains the model in two phases: the generative adversarial network (GAN) is only trained in the first phase, and in the second phase, the GAN is frozen and only the encoder is trained. The AE’s structure is formed by the generator acting as a decoder, together with the encoder. BeatGAN [22] is an algorithm for reconstructing temporal data based on generative adversarial networks with the AE as the generator, and uses adversarial regularization to further improve the model performance. DAEMON [23] uses the VAE as a generator and uses two discriminators as regularization terms in the model to avoid the problem of model overfitting. MAD-GAN [24] attempts to capture the temporal correlation of time-series data distributions using LSTM-RNN as the underlying framework for GAN networks. In TadGAN [25], the authors pointed out that the original formulation using standard adversarial loss suffers from gradient instability and pattern collapse, and introduced cycle consistency loss to train the generator by minimizing the L2 paradigm of the difference between the original and reconstructed samples.
(3) **Prediction-based methods.** These methods use an autoregressive model to model time-series data and detect anomalies by comparing actual observations with model predictions. Autoregressive integrated moving average (ARIMA) [26] predicts future observations by building an autoregressive model to determine whether the current time point is an anomaly by calculating the prediction error at each time point. Hundman et al. [27] used an LSTM instead of an autoregressive model. DeepAnT [28] uses a convolutional neural network (CNN) structure for time-series prediction. MARINA [29] considers temporal correlation and spatial correlation for time-series prediction.
(4) **Transformer and its variants.** These methods use the transformer structure [1] to efficiently capture long-term dependencies and complex patterns in temporal data for anomaly detection. An anomaly transformer [2] proposes the anomaly-attention mechanism and utilizes association discrepancy for anomaly detection. Wu et al. [3] proposed an autoformer with an auto-correlation mechanism to capture time-series dependencies based on learning cycles. Fedformer [4] uses a hybrid approach to enhance seasonal trend decomposition for temporal data.

### 2.2. Multimodal Methods for Anomaly Detection

With the development of cross-domain data fusion, multimodal learning (MML) [30,31,32] has become an important research area in recent years. It aims to exploit the complementary information between different modalities to improve the performance and robustness of machine learning and artificial intelligence systems in various tasks. Traditional deep learning methods usually process data from each modality independently and then combine their feature vectors together for training. However, this simple feature-level fusion approach cannot take full advantage of the correlation and complementarity between modalities. The goal of multimodal learning is to efficiently integrate information from multiple modalities to improve the performance and effectiveness of the task.

Most of the existing multimodal methods for anomaly detection in industrial environments focus on image anomaly detection, such as multimodal industrial anomaly detection based on 3D point cloud and RGB images. Wang et al. [5] proposed a new multimodal anomaly detection method with a hybrid fusion scheme, which achieves the fusion of point cloud features and RGB features at the same location using an unsupervised feature fusion module based on the contrast loss of patches. In the field of multimodal industrial anomaly detection based on RGB images and visible images, Zhao et al. [33] proposed a multimodal image fusion method that addresses the challenges of modeling and decomposing the required modality-specific features and modality-sharing features across modal features. Zhao et al. [34] combined a CNN and a transformer in depth and proposed a multimodal multi-label recognition transformer, which can recognize multiple objects in one image simultaneously. In addition, Ju et al. [35] proposed a transformer-based model for multimodal discourse-level sentiment detection.

## 3. Proposed Method

### 3.1. Network Structure

**Figure 1:**
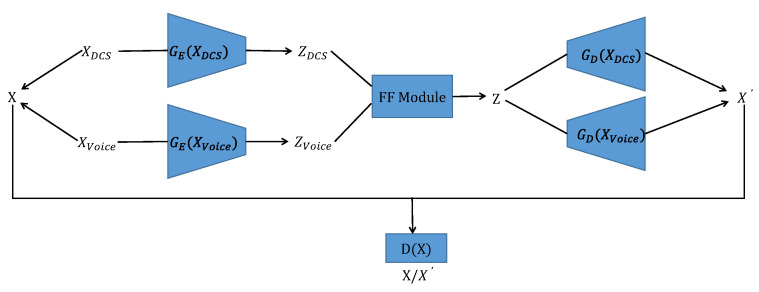
The overall architecture of MFGAN.

As shown in Figure 1, we design an MFGAN that consists of four main components: an encoder module $G_{E}\left(\cdot \right)$, a decoder module $G_{D}\left(\cdot \right)$, a feature fusion module (FF Module), and a discriminator module D(X). The encoder module consists of a distributed control system (DCS) data encoder $G_{E}\left(X_{DCS}\right)$ and an acoustic data encoder $G_{E}\left(X_{Voice}\right)$, and the decoder module consists of a DCS data decoder $G_{D}\left(X_{DCS}\right)$ and an acoustic data decoder $G_{D}\left(X_{Voice}\right)$.

Let $X_{DCS}$ be the input DCS data and let $X_{Voice}$ be the input acoustic data. They together form the input *X* of the model. The input $X_{DCS}$ is processed by $G_{E}\left(X_{DCS}\right)$ to generate the DCS hidden vector $Z_{DCS}$, and the input $X_{Voice}$ is processed by $G_{E}\left(X_{Voice}\right)$ to generate the acoustic hidden vector $Z_{Voice}$. Both $Z_{DCS}$ and $Z_{Voice}$ are processed by the FF Module to generate fused features *Z*, which are then processed by $G_{D}\left(X_{DCS}\right)$ and $G_{D}\left(X_{Voice}\right)$, respectively, to generate reconstructed data $X^{\prime }$. The discriminator *D* determines whether the input is the original data *X* or the reconstructed data $X^{\prime }$.

The overall architecture of the MFGAN combines an AE with a GAN. The MFGAN differs from other methods in its adoption of a multimodal framework that integrates a feature fusion module to employ distinct encoders and decoders for different modalities of data.

### 3.2. Data Preprocessing

#### 3.2.1. Data Format

In this work, we consider data consisting of multiple univariate time series, each of which is collected from a single data source (sensor) and arranged sequentially in chronological order. We organize such time-series data in a structured format for subsequent modeling, and use the following matrix to represent the input to the model:

(1) $$X=\left[\begin{array}{cccc} x_{11} & x_{12} & \ldots & x_{1M}\\ x_{21} & x_{22} & \ldots & x_{2M}\\ \vdots & \vdots & \; & \vdots \\ x_{N1} & x_{N2} & \ldots & x_{NM} \end{array}\right],$$

where $x_{nm}$ (1 ≤ *n* ≤ *N*, 1 ≤ *m* ≤ *M*) denotes the value of the *m*-th sensor at the timestamp $t_{n}$.

#### 3.2.2. Data Normalization

The input to the MFGAN is multidimensional time-series data, which contain information on multiple variables along different dimensions. In general, different features, such as temperature, vibration, and acoustic, have different scales, sizes, and units. Hence, without data preprocessing, it may lead to poor model performance because some features would dominate the training process due to their relatively large values. Also, the lack of data normalization may lead to model instability and hinder the model’s convergence. We use the following min-max normalization strategy to preprocess data *X*:

(2) $$X_{j}=2\cdot \frac{X_{j}-min\left(X_{j}\right)}{max\left(X_{j}\right)-min\left(X_{j}\right)}-1,$$

where $X_{j}$ is the *j*-th dimension of *X*, and $max(X_{j})$ and $min(X_{j})$ are the maximum value and the minimum value of $X_{j}$, respectively.

### 3.3. Encoder Module

Current research has shown that convolutional neural networks work better than recurrent neural networks when using AEs to process temporal data [23]. In the encoder $G_{E}\left(\cdot \right)$, we use a one-dimensional convolutional neural network to extract the features of temporal data, and also use a multi-head attention block for data processing. An attention mechanism is a technique widely used in the field of machine learning and deep learning to simulate human attentional behavior and selective attention. It can automatically focus attention on the most relevant and important parts when processing large amounts of information, thus improving the model’s ability to process critical information.

In an attention mechanism, the model learns to dynamically assign different weights to different parts of the input data to reflect their importance. This weight assignment process is based on the features and contextual information of the input data itself. By learning and adjusting the attention weights, the model selectively focuses on different features or parts, allocates more computational resources to the parts that contribute to the task, and reduces the processing of irrelevant information.

Attention mechanisms have been widely used in in-sequence modeling tasks such as machine translation, speech recognition, and temporal data analysis. In these tasks, attention mechanisms learn to assign different weights to different parts of the input sequence so that the model can generate outputs or make predictions more accurately based on contextual information. By introducing the attention mechanism, the model can better capture key information and dependencies between sequences in long sequences, allowing the model to focus its attention on key parts of the input data, thus improving the performance and robustness of the model.

**Figure 2:**
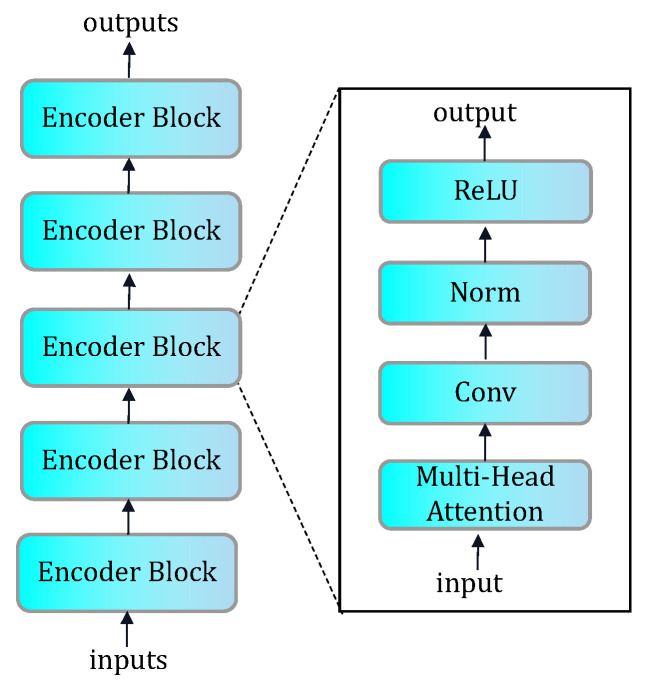
The architecture of the encoder combined with an attention mechanism.

As shown in Figure 2, our encoder consists of multiple encoder blocks, in each of which a multi-head attention mechanism helps the model focus on different parts of the input data and calculate the attention weights for each part. This allows the model to weigh different features according to their importance and thus better capture the key information of the input data. Convolutional layers, on the other hand, can extract features within local regions and capture the spatial correlation of the input data.

By stacking multiple blocks, the model is able to gradually extract higher-level, more abstract feature representations. Each block performs further feature extraction and dimensionality reduction of the input data by stacking attention mechanisms and convolutional layers, contributing to the effective learning and representation capabilities of the encoder.

### 3.4. Feature Fusion (FF) Module

Multimodal feature fusion is an important technique for anomaly detection, as it integrates features from multiple time-series data sources to improve the performance of data modeling and analysis.

Time-series data usually have temporal correlation and sequence features, such as time series, biosignals, etc. These data sources often contain rich information but also have different characteristics and dimensions. The goal of feature fusion is to merge features from different temporal data sources to form a more representative and comprehensive feature representation.

Temporal data usually involve multiple modalities, such as temperature, pressure, image, acoustic signal, etc. Multimodal fusion methods fuse features from different modalities to capture the complementarity and correlation between data from different modalities, and hence create a richer and more characterized feature representation of the data to support subsequent data modeling and analysis tasks such as classification, regression, and anomaly detection. By properly choosing and designing feature fusion methods, the information in time-series data can be better utilized to improve the performance and generalization of the model.

**Figure 3:**
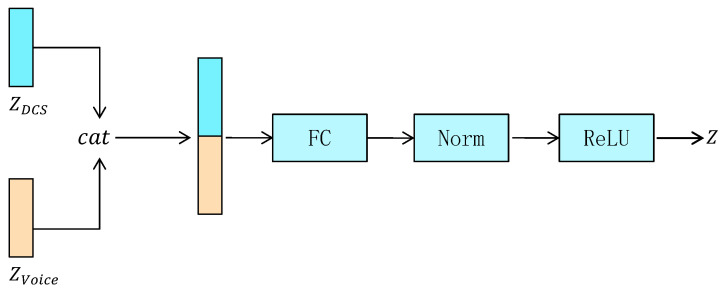
The architecture of the multimodal feature fusion module.

Therefore, we design a feature fusion module, which aims to stitch and fuse the implicit features extracted from the encoders of DCS data and acoustic data, and make full use of the correlation between the two data sources to fuse and complement each other. The feature fusion module is defined as follows:

(3) $$Z=ReLU\left(Normalization\left(w\cdot \mathrm{cat}(Z_{DCS},Z_{Voice})+b\right)\right),$$

where $Z_{DCS}$ denotes the hidden features of the DCS data generated by $G_{E}\left(X_{DCS}\right)$, $Z_{Voice}$ denotes the hidden features of the voice data generated by $G_{E}\left(X_{voice}\right)$, cat denotes the splicing operation, which concatenates $Z_{DCS}$ and $Z_{Voice}$ dimension-wise to form a larger vector, *w* is the weight matrix of the fully connected layer, *b* is the bias vector, Normalization denotes the normalization layer, ReLU denotes the activation layer, and *Z* denotes the fused features. The structure of the multimodal feature fusion module is illustrated in Figure 3.

In the feature fusion module, we first concatenate the latent features of the DCS and voice data to form a high-dimensional feature, which is then passed as input to a fully connected layer for feature fusion. The fully connected layer learns weights and bias parameters, and performs non-linear mapping and combination to fuse features, generating a more representative feature representation. Finally, the features are normalized and passed through an activation layer to form the ultimate fused feature denoted by *Z*. The fused features *Z* generated by the feature fusion module are passed to two decoders for the reconstruction of DCS data and acoustic data, respectively. The decoders re-map the fused features back to the original data space through inverse transformation to reconstruct the original data.

### 3.5. Decoder Module

In traditional AEs, the role of the decoder is to transform learned features into a reconstruction of the original input data by learning the high-level implicit feature representations extracted by the encoder and decoding them through inverse operation.

We employ an FF Module behind the output of $G_{E}\left(\cdot \right)$, which fuses features from different layers to extract a richer feature representation. In this case, the role of $G_{D}\left(\cdot \right)$ is to reconstruct the original input data based on the fused features generated by the FF module. The decoder $G_{D}\left(\cdot \right)$ receives the fused feature representation from the feature fusion module and reconstructs the original data using inverse operation.

The overall architecture of $G_{D}\left(\cdot \right)$ is the opposite of $G_{E}\left(\cdot \right)$, except that we use a one-dimensional transposed convolution as the base structure of $G_{D}\left(\cdot \right)$, which decodes the encoded hidden features into data with the same shape as the original input. Note that we do not use a multi-head attention layer in $G_{D}\left(\cdot \right)$ because it is mainly used to capture the correlation and important features in the input data. Using a multi-head attention block in $G_{E}\left(\cdot \right)$ can improve the representation of the input data. However, in the decoding stage, our goal is to reconstruct the original input data based on the hidden representation generated by $G_{E}\left(\cdot \right)$. Using a multi-head attention layer to process hidden layer features may introduce a certain degree of nonlinear transformation and feature combination, which may make the model less interpretable and even increase the risk of model overfitting. Also, it would increase the model’s complexity and redundancy due to the low dimensionality of the hidden features. Without using a multi-head attention layer, we can simplify the structure of the model and reduce the number of computations and parameters, thus improving the efficiency and training speed of the model.

### 3.6. Discriminator Module

We use a generative adversarial network as the regularization term of the AAE in the MFGAN to avoid an excessive number of parameters and overfitting of the model. The generator and discriminator continuously improve their performances during the adversarial training process.

We use a convolutional neural network as the basic structure of the discriminator D(·). During the training process, the generator tries to generate synthetic data as realistically as possible to fool the discriminator, while the discriminator tries to improve its accuracy to distinguish between real and synthetic data.

According to Equation (1) of generative adversarial networks [16], the optimization function of the GAN network is as follows:

(4) $$L_{GAN}=E_{x∼p_{data}\left(x\right)}\left[logD\left(x\right)\right]+E_{z∼p_{z}\left(z\right)}\left[logD\left(G\left(z\right)\right)\right],$$

where D(·) denotes the discriminator model, G(·) denotes the generator model, *x* denotes the true sample, *z* denotes the hidden noise sample, $x∼p_{data}\left(x\right)$ denotes the distribution of the true sample, $z∼p_{z}\left(z\right)$ denotes the prior distribution of the hidden variable, and *E* denotes the expectation.

The discriminator D(·) attempts to minimize the following loss function:

(5) $$L_{D}=-\frac{1}{n}\sum_{i}^{n}logD\left(x_{i}\right)+log\left(1-D\left(G\left(x_{i}\right)\right)\right),$$

where *n* denotes the number of samples, and $x_{i}$ denotes the *i*-th sample.

The generator G(·) tries to minimize the following loss function:

(6) $$L_{G}=\frac{1}{n}\sum_{i}^{n}log\left(1-D\left(G\left(x_{i}\right)\right)\right).$$

The pseudocode of the model in the training phase is provided in Algorithm 1.
**Algorithm 1** Training algorithm1:Initialize network parameters $\theta_{G}$, $\theta_{D}$;2:**for** each training iteration **do**3:    Sample {$X_{1}$, *…*, $X_{m}$} a batch from the normal data;4:    Generate {$Z_{DCS_{1}}$, *…*, $Z_{DCS_{m}}$}, {$Z_{Voice_{1}}$, *…*, $Z_{Voice_{m}}$} by $G_{E}$;5:    Generate integrated features {$Z_{1}$, *…*, $Z_{m}$} by the FF Module;6:    Generate {$x_{1}^{\prime }$, *…*, $x_{m}^{\prime }$} by $G_{D}$;7:    Compute $L_{D}$ by Equation (4);8:    $\theta_{D}$←$\theta_{D}+\alpha \nabla_{\theta_{D}}\left(L_{D}\right)$;9:    Compute $L_{G}$ by Equation (5);10:   $\theta_{G}$←$\theta_{G}+\alpha \nabla_{\theta_{G}}\left(L_{G}\right)$;11:**end for**

## 4. Performance Evaluation

We use both distributed control system (DCS) data and acoustic data collected from a roller press at a cement plant in Huizhou, Guangdong, China to evaluate the performance of the MFGAN for anomaly detection. We implement the algorithm using PyTorch (v1.7.1, Python 3.6.5) and run all experiments on a PC box of Windows 11 Home Edition with an Intel(R) Core(TM) i5-12500H@3.10 GHz CPU, NVIDIA 3060 6 GB GPU, and 16 GB RAM.

### 4.1. Datasets

To collect data for performance evaluation, we install vibration, temperature, and acoustic sensors on the relevant parts of the roller press at the cement plant in Huizhou, Guangdong, China, and use the DCS data and acoustic data collected by the sensors as our anomaly detection dataset. The sampling frequency of the roller press DCS data is once every second, the size of the normal dataset is 1,560,320, the size of the abnormal dataset is 173,120, and the number of features in the data is eight. The features of the DCS data are shown in Table 1.

We sample a segment of acoustic data with a duration of 25 ms every second, and the sampling rate of the acoustic data is 20 kHz, i.e., 20,000 samples per second. There are 4876 segments of normal data and 1321 segments of abnormal data in the acoustic data.

### 4.2. Evaluation Metrics

Since anomaly detection is formulated as a classification problem, we assess the performance of the MFGAN and the baselines in terms of the metrics commonly used for classification evaluation, i.e., precision (Pre), recall (Rec), accuracy (Acc), and F1-score (F1) on the test dataset.

Precision is a metric used to assess the accuracy of the positive predictions made by a model, and is defined as the number of true positive (*TP*) predictions divided by all claimed positives including both true positives and false positives (*FP*). In mathematical terms, precision is calculated as:

(7) $$Precision=\frac{TP}{TP+FP}.$$

Recall, also known as sensitivity or true positive rate, is a metric used to assess the ability of a model to capture all relevant instances of a particular class. It is defined as the number of true positive (*TP*) predictions divided by all real positives, including both true positives and false negatives (*FN*). In mathematical terms, recall is calculated as

(8) $$Recall=\frac{TP}{TP+FN}.$$

Accuracy is a metric used to evaluate the performance of a classification model, measuring the proportion of samples correctly predicted by the model across the entire dataset, calculated as:

(9) $$Accuracy=\frac{TP+TN}{TP+FP+TN+FN},$$

where *TN* denotes the number of true negatives. Note that accuracy considers both false alarms and missed detections.

The *F*1 value is the harmonic mean of precision and recall, calculated as:

(10) $$F1=\frac{2\cdot Precision\cdot Recall}{Precision+Recall}.$$

### 4.3. Baseline Methods

We compare the MFGAN with the following algorithms:

- AE [36]. The autoencoder is a classical reconstruction-based anomaly detection method with the same network structure as G(·). The parameters used in the AE are the same as those in the MFGAN.
- VAE [12]. A variational autoencoder, which combines an autoencoder and variational inference, can learn the underlying distribution of the data and generate new samples that are similar to the original data. The parameters used in the VAE are the same as those in the MFGAN.
- BeatGAN [22]. BeatGAN is a reconstruction-based model that uses adversarial generation methods to reconstruct the data. The network structure of its generator and discriminator is similar to that of the MFGAN. The parameters used in BeatGAN are the same as those in the MFGAN.
- TimesNet [15]. It converts a one-dimensional time series into a set of multi-period, two-dimensional tensors by Fourier transform, and then extracts local features for anomaly detection using a classical method in the image field. Since the TimesNet model has a different architecture than AE, VAE, BeatGAN, and MFGAN, it uses default parameters.

### 4.4. Comparative Experiments

We first describe the implementation details of our approach, including the model itself and the parameters used in the model.

#### 4.4.1. Implementation Details

Table 2 shows the relevant parameters of the model used in our experiments.

In $G_{E}\left(\cdot \right)$, we use five layers of one-dimensional convolution and five layers of multi-head attention blocks. The size and number of convolution kernels for each layer of one-dimensional convolution are 32(4/2/1)-64(4/2/1)-128(4/2/1)-256(4/2/1)-512(10/1/1), where 32(4/2/1) means that the number of filters is 32, the size of the filter is 4, the stride is 2, and the padding is 1.

In the encoder block, we use LeakyReLU as the activation function, with negative_slope set to be 0.2. LeakyReLU is a variant of Rectified Linear Unit (ReLU), which, compared with the traditional ReLU function, can be used for training deep neural networks to mitigate the gradient disappearance problem and better handle negative inputs. It can also produce smoother activation outputs in some cases.

In $G_{D}\left(\cdot \right)$, we use a five-layer, one-dimensional transposed convolution with the opposite structure of $G_{E}\left(\cdot \right)$. The structure of D(·) is similar to that of $G_{E}\left(\cdot \right)$, but without the multi-head attention layer.

#### 4.4.2. Experimental Results

We compare our proposed model with state-of-the-art multivariate time-series models for anomaly detection on the aforementioned dataset in terms of precision, recall, and F1-score.

Moreover, to ensure that the performance comparison between different models is more meaningful in terms of statistical criteria, we adopt the widely used *k*-fold cross-validation method, with *k* set to be five in our experiments. This method not only helps reduce performance fluctuations due to uneven data distribution or noise, but also improves the reliability and reproducibility of the experimental results.

For each comparative method, we conduct experiments on five disjoint data subsets, measure the performance on each subset, and calculate the average performance. In addition, we calculate the standard deviation of each performance metric to evaluate the robustness of each method on different subsets. The experimental results are provided in Table 3. These results show that the MFGAN outperforms the existing models in most of the cases. Note that a smaller standard deviation reflects a more stable performance, while a larger standard deviation indicates that the performance suffers from a higher level of variation across different data subsets. These experiments are designed and conducted to compare the performance between different methods in terms of statistical criteria, thus providing us with a deeper insight into their performance robustness.

AEs and VAEs are two common neural network models used for unsupervised learning and data generation, and they are also classical methods for reconstruction-based anomaly detection. As shown in Table 3, the VAE performs better with an F1 score 3.08% higher than the AE on our dataset because the VAE has a more powerful generative capability compared to the AE. VAEs can generate more realistic samples as it takes into account the distribution of hidden variables when generating samples. This makes VAEs perform better at data reconstruction and generation tasks. However, AEs and VAEs are less effective than BeatGAN, because they do not counter the regularization term and the quality of data sample reconstruction is relatively poor.

BeatGAN is a classical reconstruction-based anomaly detection method that combines an AE and a GAN, and it improves its F1 score by 7.14% and 4.06% compared with the AE and VAE, respectively, due to the addition of an adversarial regularization term that improves its robustness and generalization ability. This not only improves the quality of the AE’s reconstruction of samples but also helps prevent the model from overfitting the training data. However, BeatGAN does not consider multimodal feature fusion, although it also incorporates a generative adversarial network in the model.

TimesNet analyzes temporal data changes from a new multi-period perspective, extends one-dimensional temporal data to a two-dimensional space, and then reconstructs the samples by extracting features using two-dimensional convolution using the classical model in the visual field for anomaly detection. TimesNet has a suboptimal performance and improves its F1 score by 13.73% compared with BeatGan. However, it does not consider multimodal feature fusion and is unable to capture the correlation between modalities. Also, since some of the modal information may be neglected, it may lead to the loss of information, thus affecting the performance of the model.

The MFGAN proposed in this paper outperforms all other models in comparison: it improves the F1 score by 5.37% over TimesNet and by 19.1% over BeatGAN, due to the use of a multimodal feature fusion mechanism.

The model fuses rich information obtained from different sensors or data sources to alleviate the deficiencies and limitations of single modal data. When the data of one modal are corrupted or contain significant noise, the data of other modals play a complementary and corrective role to improve the accuracy and stability of anomaly detection. Meanwhile, multimodal feature fusion provides more comprehensive features. By fusing features of different modalities together, richer and more discriminative features can be obtained. This helps improve the performance of the anomaly detection model and enables it to distinguish between normal and abnormal samples more effectively.

#### 4.4.3. Time Efficiency

**Figure 4:**
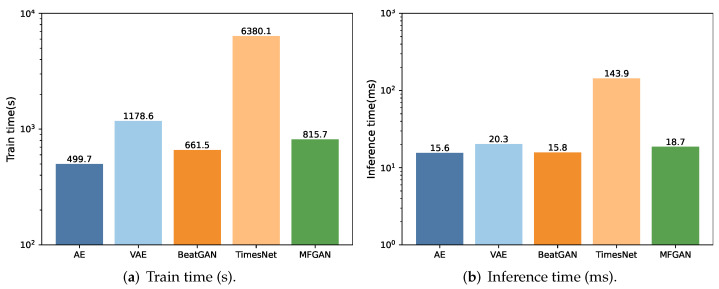
The comparison of efficiency.

In anomaly detection, apart from accuracy, model complexity and time efficiency are also important for assessing model performance. Particularly, in scenarios that require frequent model training, the model’s training time becomes pivotal. We conduct a comprehensive assessment of the training and inference times for each method, as plotted in Figure 4.

The results show that TimesNet takes significantly longer for training compared with the AE, VAE, BeatGAN, and MFGAN, due to its exceptionally complex model structure. TimesNet not only incorporates operations such as fast Fourier transform, but also stacks numerous complex networks in image domains such as InceptionNet to extract features.

In contrast, the training times for the AE, VAE, BeatGAN, and MFGAN are comparable. We observe that the AE has the shortest training time due to its simplest model structure. BeatGAN’s training time is slightly longer as it introduces the GAN on top of the AE, increasing the model’s complexity. The MFGAN takes longer for training than BeatGAN and the AE since it introduces additional attention and feature fusion modules, further increasing the complexity. The VAE’s training time is even longer than that of the MFGAN because it introduces probability distributions in the encoder and decoder. Compared with the AE, the VAE requires modeling and sampling of the latent space, which increases computational workload and time consumption during training.

The AE and BeatGAN have similar inference times since the GAN in BeatGAN is primarily utilized during the training process. During inference, it solely relies on the generator to assess anomalies, hence maintaining a consistent inference time. On the other hand, the MFGAN takes a slightly longer time for inference due to the additional attention and feature fusion modules in the model, which incur additional computational overhead during inference. However, the VAE has a longer inference time as it involves complex probability distribution inference within its encoder and decoder, incurring more computational overhead during inference. TimesNet has the longest inference time due to the most complex model structure, integrating various intricate operations.

These results indicate that: (i) compared with conventional methods, the MFGAN has a higher model complexity but its training and inference times remain within an acceptable range; (ii) the MFGAN’s training and inference speed is significantly faster than that of complex networks such as TimesNet.

### 4.5. Ablation Study

We conduct ablation experiments to further demonstrate the effectiveness of the proposed multimodal feature fusion module, the inclusion of an attention mechanism in the AE and using a GAN architecture. Similar to the above experiments for performance comparison, we also employ a 5-fold cross-validation approach to ensure that the performance measurements in the ablation experiments are meaningful in terms of statistical criteria. The experimental results of average performance and standard deviation are provided in Table 4.

As shown in Table 4, when we remove the discriminator in the MFGAN, its F1 score decreases by 9.56%, which validates the effectiveness of adopting the architecture of the GAN and introduces the discriminator for adversarial regularization in the MFGAN. Adversarial regularization not only helps the generator learn a more accurate data distribution but also further improves the reconstruction quality of normal data, enabling the model to achieve a better performance in anomaly detection. Meanwhile, we observe that the recall of the model increases by 2.67% after removing the discriminator. Our analysis, together with the confusion matrix for ablation experiments, reveals that removing the discriminator may have led to a better performance of the model in terms of capturing anomalies. However, as the model tends to label more samples as anomalies, it may increase the number of false positives, which reduces the precision and ultimately results in a decrease in the F1 score by 9.56%.

When we remove the attentional mechanism used in the MFGAN, its F1 score decreases by 11.72%, which demonstrates the effect of the attentional mechanism in the MFGAN on the processing of time-series data. This is because the attentional mechanism allows the model to autonomously focus on the information of different time steps, which improves the model’s ability to model time-series data. It provides different weights for the information at each time step in the sequence, allowing the model to focus more on the parts that are the most important to the task at hand, thus improving the model’s performance.

When we remove the FF Module used in the MFGAN, its F1 score decreases by 13.56%, which validates the effectiveness of fusing multimodal information in anomaly detection. The multimodal feature fusion module proposed in this paper fuses data from multiple modalities to fully exploit the information in the data and improve the accuracy of anomaly detection. This fusion approach improves the generalization ability and robustness of the model, and makes the model suitable for a variety of practical applications.

When we simultaneously remove the attentional mechanism, FF Module, and discriminator used in the MFGAN, the model performance decreases significantly, especially with a reduction in the F1 score by 25.99%. Combining the above results, we conclude that, for multimodal anomaly detection, the proposed components, such as the FF Module, discriminator, and attentional mechanism, play an important role in the overall model performance.

## 5. Conclusions and Future Work

The overarching achievement of our work lies in the development of a novel scheme to address the challenge of multimodal time-series anomaly detection in the industrial domain. By combining the AAE with a multimodal feature fusion module, it presents an effective solution to anomaly detection using multimodal data. Extensive experiments in a real industrial setting validate the effectiveness of the proposed model. Our research findings show that integrating an attention mechanism into a traditional AE facilitates better capturing of crucial information in time-series data and dependencies among sequences, enhancing the model’s performance and robustness in reconstructing samples. Multimodal feature fusion also presents a promising avenue for time-series anomaly detection. The performance could be further improved by integrating more data sources with appropriate feature fusion mechanisms.

There are some limitations of this work. Firstly, collecting actual anomaly data in real industrial environments poses a challenge. Anomaly data in practical industrial settings tend to be relatively scarce, making it difficult to acquire a diverse set of anomalies for model training and validation. Consequently, this imposes certain restrictions on the model’s generalization ability and adaptability to real-world scenarios. Moreover, the feature fusion method in this paper does not fully consider the complexity and diversity of industrial data. This simplification might limit the model’s capability to capture complex data patterns, thereby affecting its robustness and generalization performance. Lastly, privacy and security are important issues in some industrial applications. Such issues may be considered during the model’s design and deployment phases.

In our future work, we plan to further our research and develop new methods to effectively deal with imperfect data and take measures to improve data availability, as well as to strengthen the security of the model to protect the sensitive information and private data of the plant. We will also continue our efforts to improve the interpretability of the model to meet the practical needs of industrial applications. After addressing these issues, we will deploy the model in production industrial environments to validate its effectiveness in real-world scenarios. We also plan to explore other multimodal fusion strategies and optimize the structure of our model to improve the performance.

## Figures and Tables

**Table 1:** DCS data features.

Features
Non-drive-side bearing temperature
Drive side bearing temperature
Main motor non-drive side bearing temperature
Main motor drive side bearing temperature
Main motor stator temperature
Reducer oil temperature
Gearbox shaft temperature
Vibration of reducer

**Table 2:** The parameters used by the model during training, including the number of training rounds, batch size, temporal data split size, optimizer, learning rate, and other settings.

Model Prameters	Value
Training iterations	50
Batch size	64
Sequence length	320
Step length	160
Optimizer	Adam
Momentums of Adam	0.9 ($\beta_{1}$), 0.99 ($\beta_{2}$)
Learning rate	0.0002

**Table 3:** Comparison of average performance and standard deviation between different models for anomaly detection using *k*-fold validation. The best results are highlighted in bold.

Model	F1	Pre	Rec	Acc
AE	0.7061 ± 0.0138	0.6531 ± 0.0155	0.7962 ± 0.0154	0.7284 ± 0.0109
VAE	0.7369 ± 0.0021	0.8421 ± 0.0026	0.6657 ± 0.0033	0.7685 ± 0.0018
BeatGAN	0.7775 ± 0.0069	0.7895 ± 0.0065	0.7706 ± 0.0073	0.7993 ± 0.0054
TimesNet	0.9148 ± 0.0044	0.8637 ± 0.0059	**0.9806 ± 0.0048**	0.9271 ± 0.0055
MFGAN	**0.9685 ± 0.0046**	**0.9832 ± 0.0051**	0.9569 ± 0.0057	**0.9824 ± 0.0047**

**Table 4:** The effects of attentional mechanisms (AMs), a feature fusion module (FF Module), and a discriminator in the MFGAN. The best results are highlighted in bold. 🗸 indicates that the module is used in the experiments.

AM	FF Module	Discriminator	F1	Pre	Rec
			0.7086 ± 0.0126	0.6578 ± 0.0152	0.7967 ± 0.0143
🗸		🗸	0.8329 ± 0.0053	0.7525 ± 0.0058	0.9347 ± 0.0061
	🗸	🗸	0.8513 ± 0.0059	0.8315 ± 0.0062	0.8639 ± 0.0067
🗸	🗸		0.8729 ± 0.0086	0.7802 ± 0.0091	**0.9836 ± 0.0088**
🗸	🗸	🗸	**0.9685 ± 0.0046**	**0.9832 ± 0.0051**	0.9569 ± 0.0057

## Data Availability

Restrictions apply to the availability of these data. Data were obtained from a cement factory in Huizhou, Guangdong, China and are available from the authors with the permission of the cement factory.
